# Supplementary material for: Human capital and regional disparities: Advancing accounting frameworks with education, health, and population dynamics
Source: PLoS One. 2025 Mar 6;20(3):e0315166. doi: 10.1371/journal.pone.0315166 (PMC11884694; doi:10.1371/journal.pone.0315166)
Supplement: S1 File — This file contains five figures (S1–S5) and three tables (S1–S3) related to the explanation of inclusive wealth framework, human capital dynamics, and related data sources. (DOCX) [file pone.0315166.s001.docx]

# Supporting information

# Human Capital and Regional Disparities: Advancing Accounting Frameworks with Education, Health, and Population Dynamics.

# Inclusive Wealth framework and feature of human capital

The Inclusive Wealth Index (IWI) is a global cross-country capital account that measures the sustainable welfare of countries (UNEP, 2023; Managi & Kumar, 2018; UNU-IHDP & UNEP, 2015; UNU-IHDP, 2012). Unlike traditional economic indicators such as Gross Domestic Product (GDP), which focus on short-term economic output, the IWI captures long-term changes in capital stock. It represents a theoretical paradigm shift regarding the health and potential for future human welfare within a country's socioeconomic system (Arrow et al., 2012; Dasgupta et al., 2021).

Consider a country’s wealth as encompassing all capital assets that support sustainable development across generations. The relationship between the intergenerational welfare V(t) of a closed economy and its capital base can be expressed by the following equation:

$V\left( t \right)=V\left( \underline{K}(t) \right)=\int_{t}^{\infty} \Omega(s)N(s)u\left( c\left( \underline{K}\left( s \right), s \right) \right)e^{-\delta\left( s-j \right)}ds/\Omega(t)$ (S1.1)

Where, $\underline{K}= \{M\left( t \right),H\left( t \right), R\left( t \right)\}$ represents the initial capital assets of produced, human, and natural capital, respectively (following Dasgupta, 2018, 2021, other forms such as social, financial capital, and enabling capital are assumed to be embedded within these capitals).

$\Omega(t)$ denotes the subjective probability that the social evaluator attributes to humanity surviving beyond time t

N (t) represents population at time t,

$u\left( c\left( \underline{K}\left( s \right) \right) \right)$ denotes the per capita welfare flow generated by the consumption flow $c\left( \underline{K}\left( s \right) \right)$.

$\delta$ is the discount rate of the welfare with $\delta>0$

Assuming $V\left( t \right)$is differentiable, thus we can define wealth W (the sum of the value of these capital assets) as a linear index of social well-being:

$W\left( t \right)=p_{M}\left( t \right)M\left( t \right)+p_{H}\left( t \right)H\left( t \right)+p_{R}\left( t \right)R\left( t \right)$ (S1.2)

Where, $p_{i}\left( t \right)\equiv\frac{\partial V}{\partial K_{i}}$ on the right-hand side of equation (S2) is the partial differential of the capital assets. It is defined as the accounting price or shadow price of capital assets. In addition, the price of time is expressed as $r\left( t \right)=\frac{\partial V}{\partial t}$.

Equation (S1.2) indicates a counterfactual resource allocation mechanism to achieve wealth/welfare equivalence. Wealth is equal to the synthesis of all current and future welfare flows, meaning that future welfare can be assessed by valuing the capital stock and its value. Furthermore, one can prove that a sustainable socioeconomic system should exhibit non-declining wealth over time, i.e., total non-declining welfare. There are also price and investment principles embodied in equation (S1.2), as the investment in capital should maintain the capital asset value.

According to the definition of wealth, we can also express the price of capital assets as:

$p_{i}=\frac{1}{\Omega(t)}\int_{t}^{\infty} \Omega(s)N(s)u'\frac{\partial c(\underline{K}\left( s \right))}{\partial K_{i} (t)}e^{-\rho\delta(s-t)}\mathrm{ds}$ (S1.3)

Equation (S1.3) shows the wealth/net present value (NPV) equivalence. The NPV on the right side indicates that the optimal social benefits align with the direction of the change in inclusive wealth when and only when wealth is sustainable. In the next section, we discuss the optimization of capital in a dynamic economy.

# Human Capital in a dynamic economy

To consider the Solow growth model with knowledge externalities, originally constructed by Mankiw, Romer, and Weil (1992) and applied by Klenow and Rodriguez-Clare (1997, 2004), an economy is governed by:

$Y=F(K\left( t \right),H\left( t \right),L(t))$ (S2.1)

where the production function is specified as:

$F\left( K\left( t \right),H\left( t \right),L\left( t \right) \right)=AK^{a}H^{b}(B{L)}^{1-a-b}$ (S2.2)

In this model:

Y(t) is the total output.

K(t) is the produced capital stock.

$H\left( t \right)=L\left( t \right)h^{*}(t)$ is the human capital stock.

L(t) is the total labor force.

B represents labor efficiency (real labor participation in the job market).

A is total factor productivity (TFP).

a, b, and (1-a-b) are the elasticities of produced capital, human capital, and labor, respectively.

***Change in Human Capital***

The change in human capital is expressed as:

$I_{H}\left( t \right)=\frac{dH\left( t \right)}{dt}=\emptyset\left( Y_{s}\left( t \right), s_{s}\left( t \right) \right)-\delta L\left( t \right)h^{*}(t)$ (S2.3)

where:

$Y_{s}\left( t \right),$ and $s_{s}\left( t \right)$ denote the goods and time expenditure for education, respectively.

The change in human capital per worker is:

$I_{H}\left( t \right)=\frac{\Delta H\left( t \right)}{dt}=\frac{L\left( t \right)dh^{*}\left( t \right)}{dt}+\frac{h^{*}\left( t \right)dL\left( t \right)}{dt}$ (S2.4)

The human capital per worker then is expressed as

$\frac{dh^{*}\left( t \right)}{dt}=\frac{\emptyset\left( Y_{s}\left( t \right), s_{s}\left( t \right) \right)}{L\left( t \right)}-h^{*}\left( t \right)[\delta+n(t)]$ (S2.5)

Where, $n(t)=\frac{\mathrm{dL}\left( t \right)}{L\left( t \right)\mathrm{dt}}$ is the labor population growth rate,

***Steady State Condition***

Under steady state, the human capital per worker $h^{*}\left( t \right)$should be constant, meaning $\frac{dh\left( t \right)}{dt}=0$. Thus, we obtain:

$h^{*}\left( t \right)=\frac{\emptyset\left( Y_{s}\left( t \right), s_{s}\left( t \right) \right)}{L\left( t \right)\left( \delta+n(t) \right)}$ (S2.6)

**Current Accounting Price of Human Capital**

The accounting price (or shadow price) of human capital in the context of the dynamic economic growth model can be derived from the Hamiltonian in a dynamic optimization problem. This price represents the marginal value of an additional unit of human capital in terms of its contribution to the overall objective, such as maximizing utility or output:

$\lambda_{H}\left( t \right)=N(t){u(t)}^{'}\frac{\partial F\left( K\left( t \right),H\left( t \right), BL(t) \right)}{\partial H \left( t \right)}$ (S2.7)

The accounting price of human capital reflects the marginal value of human capital in production. Next, we discuss the practical accounting of human capital with consideration of the population dynamic.

# Circuit method of the life expectancy estimation

The estimation for **School Life Expectancy (SLE)** from life tables utilizes the classical **Sullivan prevalence-based method**, initially introduced by **Stockwell and Nam (1963)** and later followed by **Land et al. (1989, 1993)**. The method converts mortality rates into probabilities of dying using the **LIFTB function** in **Mortpak** (United Nations, 1988, 2013a). The mortality $\mu_{x}^{n}$​ for age group x can be converted to probabilities of dying $q_{x}^{n}$​ as follows:

$\left( 1-S_{x}^{n} \right)=q_{x}^{n}=\frac{n*\mu_{x}^{n}}{1-\left( n-A_{x}^{n} \right)*\mu_{x}^{n}}$. (S3.1)

In equation (5), $S_{x}^{n}$ is the survival function for person age x lives for n years. $A_{x}^{n}$ refers the average number of years lived between ages x and x + n by those dying in the interval. Denote $l_{x}$ as the Age-specific survival rate to indicate the number of livings at the beginning of the age interval (generally set at 100,000). Then there is

$l_{x}=l_{x}(1-q_{x}^{n})$. (S3.2))

$d_{x}^{n}=l_{x}-l_{x+n}$. (S3.3) $L_{x}^{n}=A_{x}^{n}l_{x}-(n-A_{x}^{n})l_{x+n}$. (S3.4) Where $L_{x}^{n}$ indicate the number of years of life lived by the cohort within the indicated age interval (x, x+n). By summing up $L_{x}^{n}$ from age x to the end, the number of person-years lived at age x, and older is $T_{x}=\sum_{a=x}^{\infty} L_{a}^{n}$. Then the life expectancy at age x (in years) $e_{x}$ is given by

${LE}_{x}=\frac{T_{x}}{l_{x}}.$ (S3.5) Similarly, denote $s_{x}$ as the school enrollment of age group x, then the number of years of schooling by the cohort within the indicated age interval (x, x+n) is ${sL}_{x}^{n}=s_{x}*L_{x}^{n}$And the number of person-years alive and in school at age x and older is${sT}_{x}=\sum_{a=x}^{\infty} sL_{a}^{n}$.

Then the school life expectancy (SLE) is calculated as

${SLE}_{5}=\frac{{sT}_{5}}{l_{5}}$. (S3.6)

The LPE (Life Participation Employment) method was first formalized in a 1983 paper by Brookshire and Cobb and later explained by Baker and Seck (1987). This method estimates work-life probability by considering three key probabilities:

L: The probability that an individual in a demographic category will be alive in any future year.

P: The probability that the individual, if alive, will participate in the labor force (either working or actively seeking work).

E: The probability that the individual will be employed if alive and participating in the labor force.

The product of these three probabilities gives the work-life probability, or the conditional probability that an individual will be alive, a labor force participant, and employed in any future year.

For each age group, we use the number of people who survived the current year as a baseline for future survival probabilities, rather than the initial population. By substitute $s_{x}$ to the labour participation rate $w_{x}$The same formulas can be applied to calculate work life expectancy as:

${WLE}_{x}=\frac{{wT}_{x}}{l_{x}}$. (S3.7) Where denote ${wT}_{x}$ as the number of person-years alive and working at age x and older.

Under the assumption of a uniform distribution of deaths, the Curtate Life Expectancy method (also known as the integer age method) is simpler to calculate. It is commonly used when high precision is not required or when detailed data on the exact timing of deaths within the year is unavailable. The Curtate method assumes that all deaths at a specific age occur exactly at the end of that year, which can result in slight underestimates of life expectancy because it does not account for additional time lived by individuals who die within the year.

In contrast, the Complete Life Expectancy method provides finer estimations by accounting for the exact timing of deaths each year. While the Curtate method is less precise, it is still suitable for broader demographic analyses and situations where data constraints exist. For our analysis, we use the Curtate method for life expectancy estimation, acknowledging that it may slightly underestimate life expectancy due to its simplified assumptions.

# Adjustment for the education attainment data

Obviously to calculate SLE needs the cohort school enrolment rates $s_{x}$. The UIS data only have school enrollment rates for different education levels and consequently, these data need to convert to $s_{x}$ for age groups. For this aim, the first step is to calculate the total population of the corresponding enrollment age group for primary, secondary, and tertiary education. This is done by using the school entry age and duration of the education for each country to the annual age population data from the UN population division. Then the number of enrolments for each year is estimated by assuming the number of students is evenly distributed in grades for the same education level. The intake rate of grade 1 is applied for checking the consistency. After that, by dividing the total number of students by the total population of the age group, the school enrolment rates $s_{x}$ are calculated. The $s_{x}$ is calculated in four age groups: 5-9, 10-14, 14-19- and 20-24-years old. Here, ore-primary education is not included in enrolment calculations. Population above 25-year-old is considered to have completed schooling.

On the other hand, the labour force participation rates $w_{x}$ can be calculated by labour force and population information collected from the ILO labor force database. $x$ refers to the age group from 15- to 69-year-old (11 groups), and the population over 70 is assumed to no longer in the labour market). The WLE is initiated from age 15 to 69, but the value of SLE determines the actual initial age of WLE for each country.

The SLE estimation applies the abridged life table from the United Nations population statistics. This life table is at five-year intervals. Since the enrolment, labour participation enrolment, and population figures are annual, the annual mortality rates are interpolated to calculate the yearly SLE.

Although the UIS data provide enrolment data for most countries, some data are missing for certain years. Table S1 shows the adjustments made to the data for countries with missing data and the basis for these adjustments.

Table S1. Supplement data sources and reference literature for adjusting the missing data of schooling.

| **Country** | **Solution** | **Third source** |
| --- | --- | --- |
| Japan | Filled by census data from JP gov which covers 1990-2018 | Source: https://www.e-stat.go.jp/en/stat-search/database?page=1&query=E380102&layout=dataset&statdisp_id=0000010105 |
| Brazil | Filled with Borro-Lee's MYS data in 1990 as the initial point and check third literature for consistency | Glewwe, Paul, and Ana Lucia Kassouf. "The impact of the Bolsa Escola/Familia conditional cash transfer program on enrollment, dropout rates and grade promotion in Brazil." *Journal of Development Economics* 97.2 (2012): 505-517. |
| Serbia |  | https://education.stateuniversity.com/pages/1322/Serbia-EDUCATIONAL-SYSTEM-OVERVIEW.html |
| Singapore |  | S.K. Lee, C.B. Goh, B. Fredriksen & J.P. Tan (Eds.), Toward a Better Future: Education and Training for Economic Development in Singapore since 1965 (pp. 149-166). |
| Yemen |  | http://documents1.worldbank.org/curated/en/664561468345850280/pdf/340080REVISED010Box334058B01PUBLIC1.pdf |
| Dominican Republic | Filled through third literature source | Latin America and the Caribbean: Selected Economic and Social Data, Volumes 1995-1997, United States. Agency for International Development, Agency for International Development, 1995, the University of Wisconsin - Madison |
| Saudi Arabia |  | Al Rawaf, Haya Saad, and Cyril Simmons. "The Education of Women in Saudi Arabia." Comparative education, vol. 27, no. 3, Taylor & Francis, Ltd., 1991, pp. 287–95, http://www.jstor.org/stable/3099298. |
| Montenegro |  | Filled data of 1990 by Borro-Lee's data of Serbia Filled data of 1996 by Silova, Iveta, and Cathryn Magno. "Gender equity unmasked: democracy, gender, and education in Central/Southeastern Europe and the Former Soviet Union." Comparative Education Review 48.4 (2004): 417-442. |
| Turkmenistan |  | Silova, Iveta, and Cathryn Magno. "Gender equity unmasked: democracy, gender, and education in Central/Southeastern Europe and the Former Soviet Union."  *Comparative Education Review* 48.4 (2004): 417-442. Huisman, Jeroen, Anna Smolentseva, and Isak Froumin. 25 Years of Transformations of Higher Education Systems in Post-Soviet Countries: Reform and Continuity. Palgrave Studies in Global Higher Education. Palgrave Macmillan. Available from: Springer Nature. One New York Plaza, Suite 4600 New York, NY 10004, 2018. |
| Bosnia and Herzegovina |  | Filled data of 1990 by Borro-Lee's data of Croatia Filled data of 1996 by Silova, Iveta, and Cathryn Magno. "Gender equity unmasked: democracy, gender, and education in Central/Southeastern Europe and the Former Soviet Union." Comparative Education Review 48.4 (2004): 417-442. |
| Korea (Democratic People's Republic of) | No adjustment | No data before 2000 |
| Curaçao |  | Population and society are relatively stable |
| Aruba |  | Population and society are relatively stable |
| Puerto Rico |  | Population and society are relatively stable |
| Somalia |  | Population and society are relatively stable |
| Cayman Islands | Originally did not calculate HC because of lacking other data (life table, labor, GDP, Wage). Excluded from EYS estimation | |
| Gibraltar |  |  |
| Liechtenstein |  |  |
| Nauru |  |  |
| Sint Maarten (Dutch part) |  |  |
| Micronesia (the Federated States of) |  |  |
| Marshall Islands |  |  |
| Palau |  |  |
| Timor-Leste |  |  |
| South Sudan |  |  |
| Sudan |  |  |
| Liberia | Second Civil War and the data after 2011 were missing, Excluded | |
| Libya |  |  |

# Country classification

Table S2 lists the geographical groupings of the 165 countries by World Bank groupings. We treat high-income countries and G20 countries as separate groups rather than by geographical region.

**Table S2 The classification of countries by region**

| **Region classification** | **Country/Economy** |
| --- | --- |
| South Asia | Afghanistan Bangladesh Bhutan Sri Lanka Maldives Nepal Pakistan |
| Europe & Central Asia | Albania Azerbaijan Armenia Bosnia and Herzegovina Belarus Georgia Kazakhstan Kyrgyzstan Moldova, Republic of Serbia Tajikistan Turkmenistan Ukraine North Macedonia Uzbekistan |
| Middle East & North Africa | Algeria Djibouti Iran (Islamic Republic of) Iraq Jordan Libya Morocco Syrian Arab Republic Tunisia Egypt Yemen |
| Sub-Saharan Africa | Angola Botswana Burundi Cameroon Cabo Verde Central African Republic Chad Congo Congo, Democratic Republic of the Benin Ethiopia Eritrea Gabon Gambia Ghana Guinea Côte d'Ivoire Kenya Lesotho Liberia Madagascar Malawi Mali Mauritania Mauritius Mozambique Namibia Niger Nigeria Rwanda Sao Tome and Principe Senegal Sierra Leone Somalia Zimbabwe Sudan Eswatini Togo Uganda Tanzania, United Republic of Burkina Faso Zambia |
| Latin America & Caribbean | Bolivia (Pluractional State of) Belize Colombia Costa Rica Cuba Dominican Republic Ecuador El Salvador Guatemala Guyana Haiti Honduras Jamaica Nicaragua Panama Paraguay Peru Suriname Venezuela (Bolivarian Republic of) |
| East Asia & Pacific | Myanmar Cambodia Fiji Korea (Democratic People's Republic of) Lao People's Democratic Republic Malaysia Mongolia Vanuatu Papua New Guinea Philippines Viet Nam Thailand Samoa |
| High income | Bahamas Bahrain Barbados Brunei Darussalam Chile Hong Kong Iceland Israel Kuwait Macao Oman New Zealand Norway Qatar Singapore Switzerland Trinidad and Tobago United Arab Emirates Uruguay |
| G20 | Argentina Australia Austria Belgium Brazil Bulgaria Canada China Croatia Cyprus Czechia Denmark Estonia Finland France Germany Greece Hungary India Indonesia Ireland Italy Japan Korea, Republic of Latvia Lithuania Luxembourg Malta Mexico Netherlands Poland Portugal Romania Russian Federation Saudi Arabia Slovakia Slovenia South Africa Spain Sweden Turkey United Kingdom of Great Britain and Northern Ireland United States of America |

**Table S3: The relationship between the human capital and the economic development.**

|  | (1) | (2) | (3) | (4) |
| --- | --- | --- | --- | --- |
| VARIABLES | GDP (per capita) | GDP (per capita) | GDP (per capita) | GDP (per capita) |
|  |  |  |  |  |
| Human capital | 100.864*** | 232.571*** | 314.579*** | 99.728*** |
|  | (1.291) | (4.181) | (4.973) | (6.331) |
| Constant | 6,403.950*** | -5,467.497*** | -13,327.916*** | 6,575.219*** |
|  | (220.230) | (1,027.087) | (465.954) | (1,061.232) |
|  |  |  |  |  |
| Observations | 4,968 | 4,968 | 4,968 | 4,968 |
| R-squared | 0.551 |  | 0.455 | 0.602 |
| Model | OLS | RE | FE | BE |

Standard errors in parentheses. *** p<0.01, ** p<0.05, * p<0.1. The OLS: ordinary least square; RE: random effects; FE: fixed effects; BE: between effects
