# Supplementary material for: Human capital and regional disparities: Advancing accounting frameworks with education, health, and population dynamics
Source: PLoS One. 2025 Mar 6;20(3):e0315166. doi: 10.1371/journal.pone.0315166 (PMC11884694; doi:10.1371/journal.pone.0315166)
Supplement: Attachment — Submitted filename: Referee_Comments_PONE_D_24_23099.pdf [file pone.0315166.s004.pdf]

Inclusive Human capital Accounting for 165 Countries Reveals the Critical Needs for Sustainability Investment

General comment

This study advances the Inclusive Wealth Human Capital Account to enhance global sustainable human capital assessment research. Authors have quantitatively measure human capital across 165 countries from 1990 to 2020 by incorporating education, health, and economic participation. Overall, I think the study is well-written, the topic is well-detailed, and I enjoyed reading it. I have some comments to improve the quality of the paper.

Major comments

1. **Introduction** Authors have detailed Introduction section. There are three sub-headings in the introduction section which are not necessary to show separately. I recommend you to remove the subsection headings by keeping the original texts. In addition, I suggest the author to read and cite Yamaguchi et al. (2019), Jingyu et al. (2020) and Islam and Managi (2021) literature for further improvement of the introduction section.

2. **Method** Some paragraphs are very short (for instance, the last paragraph of the method section) and my suggestion is to merge them with relevant paragraph.

3. **Method** Pay attention on writing the equations from 1 to 7. Why you need to put a dot (.) after every equations? To write a mathematical representations, sloppiness should be fundamentally removed.

4. **Results** At this stage, the result section is based on several plots of the panel data and their trend analysis. For robustness, I recommend the authors to consider econometric models to support your results with statistical significance.

5. **Results** Figure 6 should be improved in a way that X-axis and Y-axis information are clearly observed.

6. **Discussion** As suggested for the introduction section, the sub-section headings should be removed.

7. **Conclusion** The limitations of this study should be included in the final paragraph of the conclusion section.

8. **Conclusion** Expand the discussion on the policy implications of the findings in conclusion section.

Minor comments

1. I can observe many typos and grammatical inconsistency through the manuscript. I suggest the authors to remove it fundamentally.

2. Use the abbreviation for repeatedly used terminologies, for instance; HC can be used for Human Capital.

Overall evaluation

I think this paper made an effort to identify the necessity of incorporating complex population dynamics into human capital evaluations to better understand and enhance the inter-dependencies by aiming to reduce developmental gaps worldwide. I suggest the major revision before taking the final decision of this manuscript.

References

Islam, M. and Managi, S. (2021). Global human capital: View from inclusive wealth. In *Measuring Human Capital*, pages 39–54. Elsevier.

Jingyu, W., Yuping, B., Yihzong, W., Zhihui, L., Xiangzheng, D., Islam, M., and Managi, S. (2020). Measuring inclusive wealth of china: Advances in sustainable use of resources. *Journal of Environmental Management*, 264:110328.

Yamaguchi, R., Islam, M., and Managi, S. (2019). Inclusive wealth in the twenty-first century: a summary and further discussion of inclusive wealth report 2018. *Letters in Spatial and Resource Sciences*, 12:101–111.
