# Supplementary material for: Human capital and regional disparities: Advancing accounting frameworks with education, health, and population dynamics
Source: PLoS One. 2025 Mar 6;20(3):e0315166. doi: 10.1371/journal.pone.0315166 (PMC11884694; doi:10.1371/journal.pone.0315166)
Supplement: Attachment — Submitted filename: Rebuttal Letter.docx [file pone.0315166.s005.docx]

**To Reviewer #1**

Thank you very much for your kind and beneficial comments, which have helped us in significantly improving the quality of our article. We sincerely appreciate it. We have carefully incorporated each comment to improve our manuscript.

*Reviewer #1: Title seems a sentences which should be revised.*
**[Response]** As suggested, we revised the title into “Inclusive Human capital Accounting for 165 Countries Reveals the Critical Needs for Sustainability Investment.”.

*Abstract
There is ample space to improve the language quality and writing pattern of the paper.*

**[Response]** We revised major parts of our manuscript and followed the article structure in the author guidance of PLOS ONE. Specifically, we restructured the introduction to explain the theoretical development of human capital assessment and clarify the research gap and contribution. We also revised methodology and the detailed description and discussion of the results as suggested in other comments.

We also did professional language editing to improve the quality of our manuscript.

*Introduction
This section is not according to standard academic writing. Unnecessary sub-headings and irrelevant discussion is there. Moreover, this section should be equipped with quoting the latest studies on the topic to convince the readers about the novelty and significance of the study.*

**[Response]** Thank you for your valuable feedback. We have thoroughly revised the introduction to align with standard academic writing conventions. Specifically, the following changes were made:

Removal of Unnecessary Sub-Headings and Irrelevant Discussions:

We have removed any unnecessary sub-headings and streamlined the content to ensure that the introduction flows smoothly without irrelevant discussions. The revised version focuses on providing a concise and coherent background, emphasizing the core themes of human capital (HC) and its relationship to economic growth and sustainable development.

Quoting the Latest Studies on the Topic:

To address your concern regarding the inclusion of more recent studies, we have incorporated several new references from recent empirical research, such as Liu et al. (2023), Angrist et al. (2021), Ziberi et al. (2022), Akcigit et al. (2024), and Zhang et al. (2023). These studies offer the most up-to-date insights into HC and its connection to economic growth and development. We believe this strengthens the introduction by highlighting the novelty and significance of the current research.

Focusing on the Novelty and Significance of the Study:

In the revised introduction, we have explicitly highlighted how our study builds on recent research and fills a gap in the literature by providing a comprehensive, global analysis that integrates health, education, and population dynamics into HC accounting. This addition clearly outlines the significance of our research, particularly in terms of how it updates the HC accounts of 166 countries and examines the regional variations and dynamics of HC in relation to sustainable development.

We believe that these revisions address your concerns and provide a stronger foundation for the study, aligning the introduction with academic standards and ensuring that the novelty and importance of the research are clearly communicated.

*There is absence of theoretical foundations for this study.*

**[**Response] Thank you for your insightful comment. We have strengthened the discussion of the theoretical foundations in both the introduction and methodology sections. Specifically:

Classical and Modern Human Capital Theory:

Our study is firmly grounded in well-established economic theories of human capital. The theoretical origins of HC can be traced back to Schultz (1961) and Becker (1964), who framed human capital as a key factor in economic growth. This was further developed through the endogenous growth models of Romer (1986) and Lucas (1988), which directly link human capital accumulation to long-term economic growth. These models emphasize the role of education, skills, and technology in driving economic prosperity, providing a strong theoretical basis for our analysis.

Jorgenson-Fraumeni Lifetime Income Approach:

In our methodology, we build on the well-known Jorgenson and Fraumeni (J-F) method for estimating human capital. This approach, widely recognized in economic literature, calculates human capital as the net present value (NPV) of future earnings streams. The use of the NPV model ensures that our study is consistent with wealth accounting frameworks, following the theoretical guidelines established by Dasgupta (2014, 2018) and Arrow et al. (2012).

Health and Population Dynamics in Human Capital Models:

We also incorporate recent theoretical advancements by recognizing health as a key component of human capital. This is rooted in Grossman's (1972) theory of health as a capital good that enhances productivity. Furthermore, we integrate population dynamics—including age structure, mortality rates, and migration patterns—based on the frameworks provided by Lee and Mason (2010) and Lutz and KC (2011). This ensures that our study not only considers traditional human capital components like education and skills but also accounts for the demographic and health-related factors influencing human capital.

By building on these well-established economic theories and methodologies, we believe our study has a strong theoretical foundation. We have revised the introduction and methodology sections to make these connections more explicit, and we hope this clarifies how the study is theoretically grounded.

*Absence of literature review makes the quality of the study, very poor.*

[Response] Thank you for your constructive feedback. Following the author guidance of PLOS ONE, we have now integrated a comprehensive review of the relevant literature directly into the introduction section. Specifically:

Incorporation of Recent and Relevant Studies:

The revised introduction includes references to a wide range of recent studies that address the relationship between human capital and economic growth (e.g., Liu et al., 2023; Angrist et al., 2021; Ziberi et al., 2022; Akcigit et al., 2024; Zhang et al., 2023). This review of contemporary research provides strong empirical support for the study’s focus on human capital's role in economic development.

Contextualizing Human Capital within Sustainable Development:

We have also integrated literature that explores the broader contributions of human capital to sustainable development. Key studies and global initiatives such as the World Bank’s Human Capital Index (HCI, 2018) and the United Nations Sustainable Development Goals (SDGs, 2015) are discussed to frame the importance of human capital in both economic and social contexts.

Gaps in the Literature and Contributions of the Study:

Additionally, we identify gaps in the existing literature, such as the need for a more comprehensive model that integrates health, education, and population dynamics in human capital accounting (e.g., Lim et al., 2018; Campbell and Üngör, 2020). By addressing these gaps, the revised literature review shows how our study contributes to the field by incorporating these factors into human capital estimations for 166 countries from 1990 to 2020.

We believe that these revisions have addressed the absence of a literature review and have significantly improved the quality of the study by grounding it in a thorough discussion of the most relevant and recent research.

References

Adawo, M. A. (2011). Has education (human capital) contributed to the economic growth of Nigeria?. Journal of economics and international finance, 3(1), 46.

Adelakun, O. J. (2011). Human capital development and economic growth in Nigeria. European journal of business and management, 3(9), 29-38.

Aghaei, M., Rezagholizadeh, M., & Bagheri, F. (2023). The effect of human capital on economic growth: The case of Iranâ s provinces. Quarterly Journal of Research and Planning in Higher Education, 19(1), 21-44.

Akcigit, U., Pearce, J., & Prato, M. (2024). Tapping into talent: Coupling education and innovation policies for economic growth. Review of Economic Studies, rdae047.

Arrow, K. J., Dasgupta, P., Goulder, L. H., Mumford, K. J., & Oleson, K. (2012). Sustainability and the measurement of wealth. Environment and development economics, 17(3), 317-353.

Bhargava, A., Jamison, D. T., Lau, L. J., & Murray, C. J. (2001). Modeling the effects of health on economic growth. Journal of health economics, 20(3), 423-440.

Bloom, D. E., Canning, D., & Sevilla, J. (2004). The effect of health on economic growth: a production function approach. World development, 32(1), 1-13.

Bouznit, M., Pablo-Romero, M. P., & Sánchez-Braza, A. (2023). Economic growth, human capital, and energy consumption in Algeria: evidence from cointegrating polynomial regression and a simultaneous equations model. Environmental Science and Pollution Research, 30(9), 23450-23466.

Cohen, D., & Soto, M. (2007). Growth and human capital: good data, good results. Journal of economic growth, 12, 51-76.

Dritsaki, M., & Dritsaki, C. (2024). The relationship between health expenditure, CO2 emissions, and economic growth in G7: Evidence from heterogeneous panel data. Journal of the Knowledge Economy, 15(1), 4886-4911.

Elkhalfi, O., Chaabita, R., Zahraoui, K., & El Alaoui, H. (2023). Public Spending on Human Capital and Economic Growth in Morocco. International Journal of Economics and Financial Issues, 13(4), 102-110.

Goldin, C. (2024). Human capital. In Handbook of cliometrics (pp. 353-383). Cham: Springer International Publishing.

Islam, M. and Managi, S. (2021). Global human capital: View from inclusive wealth. In Measuring Human Capital, pages 39–54. Elsevier.

Jingyu, W., Yuping, B., Yihzong, W., Zhihui, L., Xiangzheng, D., Islam, M., and Managi, S. (2020). Measuring inclusive wealth of china: Advances in sustainable use of resources. Journal of Environmental Management, 264:110328.
 Liu, D., Wang, G., Sun, C., Majeed, M. T., & Andlib, Z. (2023). An analysis of the effects of human capital on green growth: effects and transmission channels. Environmental Science and Pollution Research, 30(4), 10149-10156.

Manca, F. (2012). Human capital composition and economic growth at the regional level. Regional Studies, 46(10), 1367-1388.

Mushkin, S. J. (1962). Health as an Investment. Journal of political economy, 70(5, Part 2), 129-157.

Siddiqui, A., & Rehman, A. U. (2017). The human capital and economic growth nexus: in East and South Asia. Applied Economics, 49(28), 2697-2710.

Yormirzoev, M. (2023). Human capital and economic growth in Central Asia. Post-communist Economies, 35(6), 533-545.

Zhang, Y., Kumar, S., Huang, X., & Yuan, Y. (2023). Human capital quality and the regional economic growth: Evidence from China. Journal of Asian Economics, 86, 101593.

Ziberi, B. F., Rexha, D., Ibraimi, X., & Avdiaj, B. (2022). Empirical analysis of the impact of education on economic growth. Economies, 10(4), 89.

*Used methodology should be elaborated in more details.*
**[Response]** Thank you for your valuable comment. In response, we have elaborated on the methodology to provide greater clarity and detail. Specifically, the following enhancements were made:

Detailed Description of Population Stages and Human Capital Calculation:

We have provided a more thorough explanation of how the population is grouped into three stages (0-4 years, school life expectancy, and post-education). The methodology now clearly explains how human capital is estimated at each stage, using the Mincer equation and the Jorgenson-Fraumeni (J-F) lifetime income approach.

Inclusion of Mathematical Formulations and Their Interpretation:

We have included detailed mathematical expressions for key components, such as the Net Present Value (NPV) formula for human capital, the estimation of school life expectancy (SLE), and work-life expectancy (WLE). Each equation is followed by a clearer explanation of its significance and the assumptions behind it.

Health and Population Dynamics Integration:

We have further clarified how health and population dynamics (e.g., age structure, survival rates, job participation) are integrated into human capital accounting. This includes the use of Markov models for calculating life expectancy and participation rates, as well as how health outcomes are factored into the overall framework.

Data Sources and Processing:

To improve transparency, we have elaborated on the data sources used in the study, including data from UNESCO, ILO, and United Nations Population Statistics, and the adjustments made to standardize school enrollment and labor force participation rates across countries.

These additions provide a more comprehensive understanding of the methodology used in the study, ensuring that it is fully transparent and replicable. We believe these revisions address your concern and significantly improve the clarity of the methodological framework.

*The rationale to select the sample should be justified.*
**[Response]** Thank you for your comment. We would like to clarify that our study is not based on a sample selection process but rather on a global human capital accounting framework. The study utilizes comprehensive data from 166 economies spanning 1990 to 2020 to provide a complete and comparative analysis of human capital trends across countries and regions.

We use multiple public global databases, including those from UNESCO, ILO, and United Nations Population Statistics, and supplement these with reliable national statistics or literature where necessary to ensure comprehensive global human capital accounting. While we acknowledge that there are some limitations in the fineness of the data, particularly in tracking changes in school enrollment and job market participation, the accounting framework we employ is flexible and allows for future improvements as more refined data becomes available. Moreover, the accounting results are consistent with those from other well-known studies, such as the World Bank's human capital accounting (2020) and Lee et al.(2018), further validating our approach.

By relying on this global accounting approach, we ensure that the study captures global trends and regional variations in human capital, making it more robust and generalizable than a sample-based analysis. We have revised the manuscript to clarify this methodological approach and believe it now better addresses your concern regarding the rationale for sample selection.

*Each figure should be explained in details.*

**[Response]** Thank you for your helpful feedback. In response, we have revised the manuscript to provide more detailed explanations of each figure, ensuring that the key insights and interpretations are clearly communicated. Specifically:

Detailed Explanation of Key Findings:

We have expanded the descriptions of each figure, focusing on the main findings and their relevance to the overall analysis. For example, in Figure 1, we now provide a clearer breakdown of the regional disparities in HC growth between 1990 and 2020, emphasizing the inverse relationship between initial HC levels and growth. In Figure 2, we further explain the correlation between HC and GDP per capita and the variations observed between 1990 and 2020 in different quartiles.

Clarifying Regional Trends:

For figures that compare regional trends (e.g., Figures 3, 4, 5), we have improved the explanations by highlighting how the different components of HC—such as education, adult labor proportion, and accounting price—vary across regions. For instance, in Figure 5, we explain the complex relationship between education and remaining working life across different regions, pointing out where inverse relationships are observed and how this affects overall HC accumulation.

Improved Discussion of Relationships Between Variables:

In response to your comment, we also explained the figures showing the variables' relationships. For example, Figure 4 now includes a more detailed discussion of how changes in school life expectancy (SLE), adult population share, and remaining working years contribute to overall HC growth and how these components are related to GDP growth.

*Discussion of the topic should be linked with earlier literature.*

[**Response**] Thank you for your insightful comment. In response, we have revised the discussion section to more explicitly link our findings with earlier literature, drawing comparisons and highlighting areas of agreement or divergence.

Comparing HC and Economic Development:

We now refer to studies such as Liu et al. (2023), Angrist et al. (2021), and Cohen and Soto (2007) when discussing the discrepancy between HC growth and economic development. Our results are consistent with these earlier findings, which also show that human capital does not always lead to proportional GDP growth, particularly in lower-income regions.

Education and Non-Educational HC Components:

The revised discussion links our findings on education and non-educational HC components to prior work, including Lim et al. (2018) and the World Bank HC Index (2018). We specifically highlight how our findings on the lag in non-educational components, such as health and economic participation, align with previous studies but also show some regional variations that have been less explored in the literature.

Complex Interactions of Life Expectancy, Education, and Economic Participation:

We reference studies such as Becker (2007) and Bloom and Canning (2000) to explain the complex interactions between life expectancy, education, and economic participation, which are consistent with earlier findings. However, we also point out how our analysis reveals additional complexities in less-developed regions, where increasing life expectancy does not always result in proportional HC growth.

By incorporating these references and linking our findings more explicitly to earlier literature, we believe the revised discussion now provides a more comprehensive understanding of how our results fit within the broader academic context. These changes help to situate our contributions in the ongoing conversation about HC and economic development.

*Theoretical, practical implications are also missing. Moreover, there is need to highlight the limitations and future prospects of the study.*

**[Response]** Thank you for your valuable feedback. In response, we have revised the discussion and conclusion sections to explicitly address the theoretical and practical implications of the study, as well as to more clearly outline the limitations and future prospects. The changes are as follows:

Theoretical Implications:

In the revised discussion, we now highlight the theoretical contributions of our study, particularly how our integration of health and population dynamics into human capital (HC) accounting advances existing HC models. We discuss how the use of the Net Present Value (NPV) framework provides a more comprehensive understanding of the long-term relationship between investment in education, health, and economic outcomes, contributing to the growing body of literature on human capital and sustainable development.

Practical Implications:

We also added a section on practical implications, focusing on the policy recommendations that arise from our findings. Specifically, we emphasize the need for policymakers to adopt a holistic approach to HC development, investing not only in education but also in health and economic participation to foster more equitable and sustainable growth. This is particularly relevant for low-income regions where neglecting non-educational aspects of HC may hinder long-term development.

Limitations:

In the revised conclusion, we now explicitly acknowledge the limitations of the study, such as the reliance on life tables for estimating life expectancy, the Curtate method for estimating working life expectancy, and the fixed discount rates used in the analysis. These limitations may affect the granularity of the results, particularly regarding age-specific population dynamics, and we outline how these issues could be addressed in future research.

Future Prospects:

We also include a section on future research directions, suggesting that future studies could incorporate dynamic estimations of health, education, and economic participation across different age groups to provide more detailed insights. Additionally, we recommend expanding the dataset and conducting more granular analyses to explore regional variations in HC accumulation and their relationship to economic growth in greater depth.

These revisions ensure that the theoretical and practical implications, as well as the limitations and future prospects, are clearly articulated, addressing your concerns and enhancing the overall contribution of the study.

**To Reviewer #2**

Thank you very much for your kind and beneficial comments, which have helped us in significantly improving the quality of our article. We sincerely appreciate it. We have carefully incorporated each comment to improve our manuscript.

*Reviewer #2: Inclusive Human capital Accounting for 165 Countries Reveals the Critical Needs for Sustainability Investment

General comment
This study advances the Inclusive Wealth Human Capital Account to enhance global sustainable human capital assessment research. Authors have quantitatively measure human capital across 165 countries from 1990 to 2020 by incorporating education, health, and economic participation. Overall, I think the study is well-written, the topic is well-detailed, and I enjoyed reading it. I have some comments to improve the quality of the paper.*

*Major comments
1. Introduction Authors have detailed Introduction section. There are three sub-headings in the introduction section which are not necessary to show separately. I recommend you to remove the subsection headings by keeping the original texts. In addition, I suggest the author to read and cite Yamaguchi et al. (2019), Jingyu et al. (2020) and Islam and Managi (2021) literature for further improvement of the introduction section.*
**[Response]** Thank you for your helpful feedback. In response to your suggestions, we have made the following revisions to the introduction section:

Removal of Sub-headings:

We have removed the sub-section headings in the introduction, as recommended, while keeping the original text intact. The content now flows seamlessly as a cohesive narrative, enhancing the clarity and readability of the introduction.

Citing Additional Literature:

We have reviewed and incorporated the suggested references into the introduction as

*“…Recent empirical research has focused on country-specific analyses of HC and its relationship with economic growth (Arrow et al., 2012; Islam and Managi, 2021; Jingyu et al., 2020). This body of work provides compelling evidence that improvements in educational attainment are strongly correlated with economic development (Liu et al., 2023; Angrist et al., 2021; Cohen and Soto, 2007; Siddiqui and Rehman, 2017; Adawo, 2011; Yormirzoev, 2023; Ziberi et al., 2022; Akcigit et al., 2024; Zhang et al., 2023; Elkhalfi et al., 2023; Bouznit et al., 2023). For instance, Angrist et al. (2021), utilizing global learning data from 164 countries between 2000 and 2017, highlighted the positive effects of HC on economic growth. Similarly, regional studies have shown that the impact of education on growth varies: in East Asia, primary and secondary education have a stronger effect, while tertiary and vocational education are more impactful in South Asia (Adelakun, 2011; Adawo, 2011).”*

The studies Jingyu et al. (2020), and Islam and Managi (2021) provide valuable insights into the relationship between human capital, environmental sustainability, and economic development, which further strengthen the theoretical framework of our study. Their inclusion enriches the discussion on human capital dynamics and reinforces the importance of integrated approaches to sustainable development.

These changes have improved the introduction section, ensured a more focused and relevant presentation of the topic while aligned with the latest research in the field.

*2. Method Some paragraphs are very short (for instance, the last paragraph of the method section) and my suggestion is to merge them with relevant paragraph.*

**[Response]** Thank you for your helpful suggestion. In response, we have revised the method section by merging shorter paragraphs with the relevant preceding sections to improve the flow and cohesiveness of the text. Specifically, the last paragraph has been integrated into the appropriate section discussing data sources and the analytical framework. This ensures that the methodology is presented in a more structured and unified manner.

These changes have enhanced the clarity and readability of the method section, aligning it with your recommendations.

*3. Method Pay attention on writing the equations from 1 to 7. Why you need to put a dot (.) after every equations? To write a mathematical representations, sloppiness should be fundamentally removed.*

[**Response**] Thank you for pointing this out. We have revised the equations in the method section to ensure consistency and clarity. Specifically, we have removed the **dots (.)** following each equation, as they are unnecessary in mathematical representations. Additionally, we have carefully reviewed all equations to eliminate any sloppiness and to ensure that the formatting is precise and meets the standards for clear mathematical presentation.

These corrections help improve the professionalism and readability of the equations in the manuscript.

*4. Results At this stage, the result section is based on several plots of the panel data and their trend analysis. For robustness, I recommend the authors to consider econometric models to support your results with statistical significance.*

**[Response]** Thank you for your valuable suggestion. In response to your recommendation, we have incorporated an econometric analysis to strengthen the robustness of our results. Specifically, we have added **Table S3** to the manuscript, which presents the relationship between **human capital (HC)** and **economic development** using various econometric models, including **Ordinary Least Squares (OLS), Random Effects (RE), Fixed Effects (FE)**, and **Between Effects (BE).**

The table compares the statistical significance of **HC** in relation to **GDP per capita** across the four models. Our results show that **HC** has a statistically significant positive relationship with **GDP per capita** in all models, with significance levels of ***p<0.01** in each case. These results provide further evidence of the robustness of our findings and support the trends observed in the panel data plots.

We have also ensured that the standard errors are presented, and we report the **R-squared** values to demonstrate the explanatory power of each model. By including this additional econometric analysis, we have strengthened the statistical foundation of the results, aligning with your recommendation to improve the robustness of the study.

Table S3: The relationship between the human capital and the economic development.

|  | (1) | (2) | (3) | (4) |
| --- | --- | --- | --- | --- |
| VARIABLES | GDP (per capita) | GDP (per capita) | GDP (per capita) | GDP (per capita) |
|  |  |  |  |  |
| Human capital | 100.864*** | 232.571*** | 314.579*** | 99.728*** |
|  | (1.291) | (4.181) | (4.973) | (6.331) |
| Constant | 6,403.950*** | -5,467.497*** | -13,327.916*** | 6,575.219*** |
|  | (220.230) | (1,027.087) | (465.954) | (1,061.232) |
|  |  |  |  |  |
| Observations | 4,968 | 4,968 | 4,968 | 4,968 |
| R-squared | 0.551 |  | 0.455 | 0.602 |
| Model | OLS | RE | FE | BE |

Standard errors in parentheses. *** p<0.01, ** p<0.05, * p<0.1. The OLS: ordinary least square; RE: random effects; FE: fixed effects; BE: between effects

*5. Results Figure 6 should be improved in a way that X-axis and Y-axis information are clearly observed.*

**[Response]** Thank you for your helpful comment. In response, we have improved Figure 6 to ensure that both the X-axis and Y-axis information are clearly visible and easily interpretable. We have adjusted the axis labels to ensure they are more legible by increasing the font size and enhancing the contrast. Additionally, we have ensured that the axis titles and units are now clearly defined for better readability.

These changes improve the clarity of the figure and make the data trends easier to interpret.

*6. Discussion As suggested for the introduction section, the sub-section headings should be removed.*

**[Response]** Thank you for your suggestion. In response, we have removed the sub-section headings from the Discussion section, as you recommended. The content now flows as a unified narrative without the use of sub-headings, ensuring consistency with the structure of the Introduction section.

*“This study updates the HC account from the latest Inclusive Wealth report (Managi et al. 2024, UNEP. 2023) and presents the HC accounting approach based on the incorporation of health and population dynamics modelling. The analysis from 1990 to 2020 reveals significant disparities in HC development among 165 countries. In this section, we discussed the aforementioned results in the following three main points.*

*First, the discrepancy relationship between HC and economic development are confirmed, which is consistent with the previous studies (Liu et al., 2023; Angrist et al., 2021; Cohen and Soto 2007; Siddiqui and Rehman 2017; Adawo 2011;Yormirzoev 2023; Ziberi et al., 2022; Akcigit et al., 2024; Zhang et al., 2023; Elkhalfi et al., 2023; Bouznit et al., 2023). Our analysis reveals that although there has been significant growth in HC over the past three decades, this growth does not uniformly correspond to GDP growth. Notably, disparities are evident across different regional groupings, with a widening gap between high-income countries and the economically robust G20 nations compared to other regions. This discrepancy suggests that factors other than HC might be driving GDP growth or that HC impacts GDP more complexly than previously understood.*

*Second, The impacts of non-educational components of HC on health and economic outcomes are lag behind. While improvements in education are strongly correlated with GDP growth, the components of HC related to health and economic participation lag significantly behind, especially in less developed regions such as South Asia and sub-Saharan Africa. Mortality risks, including diseases and environmental factors such as air pollution, may be critical in these disparities. This lag suggests that improvements in education alone are insufficient to boost long-term HC growth without concomitant improvements in health and economic conditions.*

*Third, the interactions among life expectancy, education, and economic participation are complexed. The relationship between life expectancy and different dimensions of HC shows a contradiction between investment in education and lifetime returns. Increases in life expectancy do not necessarily translate into improvements in HC per capita. This contradiction is evident when increased schooling does not lead to increased economic participation or increased adult life expectancy. Such dynamics suggest that increasing years of education may not always lead to commensurate gains in economic productivity or health outcomes.*

*Fourth, there are regional variations in HC components in high income and G20 countries, the results are in line with the World Bank HC index (HCI, 2018) and Lim et al., (2018). The components of HC—education, health, and economic participation—contribute synergistically to growth. Conversely, in other regions, the sluggish advancement in health and economic participation can hinder the effectiveness of educational investments, leading to stunted HC growth. This variation underscores the necessity for integrated strategies that address all aspects of HC to foster equitable development.”*

We believe this change improves the readability and coherence of the discussion.

*7. Conclusion The limitations of this study should be included in the final paragraph of the conclusion section.*

**[Response]** Thank you for your helpful suggestion. In response, we have added a dedicated section in the final paragraph of the conclusion to explicitly discuss the limitations of the study. We highlight key constraints such as the reliance on life tables for estimating life expectancy, the use of the Curtate method for estimating work life expectancy, and the assumptions made regarding fixed discount rates. Additionally, we acknowledge that the granularity of the data is limited in certain areas, particularly regarding age-specific population dynamics and regional disparities.

By outlining these limitations, we provide a clearer context for interpreting the results and suggest areas for future research, such as expanding the data set and incorporating dynamic modeling to improve the precision of the analysis.

The revised is as follow:

*“The limitations of this study are acknowledged as follows. First, due to data availability constraints, our life expectancy estimates are based on life tables rather than survival functions and using the Curtate method and assuming that deaths are evenly distributed by age to estimate life expectancy alongside school life expectancy and working life expectancy by sex (Loichinger E. and Weber D. 2016, Land et al. 1989, 1993, Stockwell and Nam. 1962). Life expectancy calculations using the Curtate method typically have an estimated error of half a year compared to the Complete method. However, the inclusion of integrated age-specific data on schooling and labour force participation in the population ensures that our estimates more accurately reflect the consistency of education, health, and participation in the accounting system compared to previous accounts, aligning well with the analysis requirements for this investigation (refer Supporting Information S2). Furthermore, we have set both the return rate to education and the discount rate at 0.085. The School Life Expectancy (SLE) and Work Life Expectancy (WLE) are fixed to estimate the population's HC, which suffices for our cost-benefit analysis. However, dynamic estimations of health, education, and economic participation status across all age groups could provide more detailed insights into each age cohort of the population, enhancing our financial and policy analysis. Future work should expand upon this by extending the data to conduct a comprehensive modelling exercise.”*

*8. Conclusion Expand the discussion on the policy implications of the findings in conclusion section.*

**[Response]** Thank you for your insightful comment. In response, we have expanded the discussion on policy implications in the conclusion section. Specifically, we now highlight how the findings can inform policymaking in areas such as education investment, healthcare infrastructure, and economic participation. We emphasize that a holistic approach—addressing education, health, and economic participation simultaneously—will be necessary for sustainable HC development, particularly in low-income regions where non-educational aspects are often overlooked.

We also discuss how policymakers should prioritize integrated strategies to improve both the quality and access to education, while simultaneously investing in health and employment opportunities, ensuring a comprehensive development of HC that can drive long-term economic growth and social welfare.

These additional points strengthen the relevance of our findings to policy design, ensuring the study offers practical insights for achieving sustainable economic development.

The policy implications referring to the findings of the study are as follows.

*“A positive relationship between the HC and economic development is confirmed in this study based on the data derived from 165 countries, which suggests that improvement of the HC is associated with economic development and health improvement. Regarding the component of the HC, education presents a favorable effect on economic development, and the population education expectation increases, economic development follows. The results suggest that policymakers should make sufficient financial investments in educational institutions to improve the population's education and skills. However, the empirical evidence in this study showing the effect of the non-educational HC on health and the economic outcomes are time-lagged, indicating the comprehensive investment in the HC is favorable to achieving sustainable economic development in the long-term perspective.”*

*Minor comments
1. I can observe many typos and grammatical inconsistency through the manuscript. I suggest the authors to remove it fundamentally.*

**[Response]** As recommended, we conducted a thorough copyediting and revision of the manuscript to address any errors.

*2. Use the abbreviation for repeatedly used terminologies, for instance; HC can be used for Human Capital.*

**[Response]** Thank you for your suggestion; in the revision, we applied this comment and used the HC for the Human Capital.

*Overall evaluation
I think this paper made an effort to identify the necessity of incorporating complex population dynamics into human capital evaluations to better understand and enhance the inter-dependencies by aiming to reduce developmental gaps worldwide. I suggest the major revision before taking the final decision of this manuscript.*

References
Islam, M. and Managi, S. (2021). Global human capital: View from inclusive wealth. In Measuring Human Capital, pages 39–54. Elsevier.
Jingyu, W., Yuping, B., Yihzong, W., Zhihui, L., Xiangzheng, D., Islam, M., and Managi, S. (2020). Measuring inclusive wealth of china: Advances in sustainable use of resources. Journal of Environmental Management, 264:110328.
Yamaguchi, R., Islam, M., and Managi, S. (2019). Inclusive wealth in the twenty-first century: a summary and further discussion of inclusive wealth report 2018. Letters in Spatial and Resource Sciences, 12:101–111.
