# Supplementary material for: Human capital and regional disparities: Advancing accounting frameworks with education, health, and population dynamics
Source: PLoS One. 2025 Mar 6;20(3):e0315166. doi: 10.1371/journal.pone.0315166 (PMC11884694; doi:10.1371/journal.pone.0315166)
Supplement: Attachment — Submitted filename: Rebuttal_Letter_auresp_2.docx [file pone.0315166.s006.docx]

**To Reviewer #2**

Reviewer #2: Authors have taken account of the comments and modify the manuscript accordingly. I recommend the acceptance of the manuscript in PlosONE.

**We sincerely thank you for your positive evaluation of our manuscript and their recommendation for acceptance. We greatly appreciate your constructive feedback, which has helped us improve the manuscript.**

**To Reviewer #3**

Reviewer #3: The manuscript should be proofread by an English native speaker. There are very long phrases in the text, that make the paper difficult to read. For example:

"Data required for cohort enrolment probabilities are provided by UNESCO Education Statistics (UIS) (2021), which includes gross enrolment rates and enrolment rates for primary, secondary, and tertiary schooling by gender." should be rephrased as: "Data required for cohort enrolment probabilities are provided by UNESCO Education Statistics (UIS) (2021). They include gross enrolment rates and enrolment rates for primary, secondary, and tertiary schooling by gender."

**[Response]** We sincerely thank you for your detailed comments and suggestions to improve the clarity of our manuscript. In response, we have:

(1) Carefully proofread the entire manuscript and sought feedback from a native English speaker to refine the language. (Please see the attached certification of proofreading.)
(2) Revised overly long sentences for better readability, including the specific example you highlighted. The revised text now reads:

"*Data required for cohort enrolment probabilities are provided by UNESCO Education Statistics (UIS) (2021). They include gross enrolment rates and enrolment rates for primary, secondary, and tertiary schooling by gender.*"

We greatly appreciate your constructive feedback, which has significantly enhanced the quality and readability of our work.
